# Rapid and Sensitive Detection of *Listeria ivanovii* by Loop-Mediated Isothermal Amplification of the *smcL* Gene

**DOI:** 10.1371/journal.pone.0115868

**Published:** 2014-12-30

**Authors:** Yi Wang, Yan Wang, Huaqing Xu, Hang Dai, Shuang Meng, Changyun Ye

**Affiliations:** 1 State Key Laboratory for Infectious Disease Prevention and Control, National Institute for Communicable Disease Control and Prevention, China CDC, Changbai Road 155, Changping, Beijing, 102206, PR China, Collaborative Innovation Center for Diagnosis and Treatment of Infectious Diseases, Hangzhou, PR China; 2 Guiyang Medical University, Guiyang, PR China; Royal Tropical Institute, Netherlands

## Abstract

A loop-mediated isothermal amplification (LAMP) assay for rapid and sensitive detection of the *L. ivanovii* strains had been developed and evaluated in this study. Oligonucleotide primers specific for *L. ivanovii* species were designed corresponding to *smcL* gene sequences. The primers set comprise six primers targeting eight regions on the species-specific gene *smcL*. The LAMP assay could be completed within 1 h at 64°C in a water bath. Amplification products were directly observed by the Loopamp Fluorescent Detection Reagent (FD) or detected by agarose gel electrophoresis. Moreover, the LAMP reactions were also detected by real-time measurement of turbidity. The exclusivity of 77 non-*L. ivanovii* and the inclusivity of 17 *L. ivanovii* were both 100% in the assay. Sensitivity of the LAMP assay was 250 fg DNA and 16 CFU per reaction for detection of *L. ivanovii* in pure cultures and simulated human stool. The LAMP assay was 10 and 100-fold more sensitive than quantitative PCR (qPCR) and conventional PCR assays,respectively. When applied to human stool samples spiked with low level (8 CFU/0.5 g) of *L. ivanovii* strains, the new LAMP assay described here achieved positive detection after 6 hours enrichment. In conclusion, the new LAMP assay in this study can be used as a valuable, rapid and sensitive detection tool for the detection of *L. ivanovii* in field, medical and veterinary laboratories.

## Introduction

*Listeria ivanovii* (*L. ivanovii*), a Gram-positive, facultative intracellular pathogen, is one of major pathogenic species of the genus *Listeria*, which contains fifteen species that are phylogenetically related [1]–[3]. *L. ivanovii* is considered to be specific to ruminants and responsible for 15% of all animal listeriosis [4]. The important clinical manifestations of listeriosis due to *L. ivanovii* include enteritis, neonatal sepsis, and abortion but no infection of the brain [5], [6]. Human listeriosis cases involving *L. ivanovii* have been reported in susceptible individuals belonging to special at-risk groups, such as persons of advanced age, patients suffering from carcinoma and with AIDS [7]–[9]. Moreover, the *L. ivanovii* contribution to human cases may have been underestimated according to Reissbrodt *et* *al.* [10]. Thus, it is critical to develop a rapid, specific, sensitive and cost-effective assay for detection of *L. ivanovii* in both medical and veterinary diagnostic laboratories.

The traditional diagnostic method for *L. ivanovii* involves enrichment procedures in selective broths (such as Half-Fraser or Fraser), isolation on selective media and subsequent identification (biochemical or haemolysis tests) [11]. However, the culture-based method is time-consuming, labor-intensive and inefficient, making it difficult to rapidly detect *L. ivanovii* strains associated with outbreaks and sporadic cases [12]. Additionally, the identification of a *L. ivanovii* colony from other *Listeria* species grown on a *Listeria*-selective agar, such as BLAB and OXFORD, is difficult because of the frequent coexistence of *L. ivanovii* with other *Listeria* spp. such as *L. innocua* and *L. monocytogenes*, which generally outgrow *L. ivanovii* [13]. The molecular methods, such as conventional PCR and real-time PCR assays, have been used for rapidly screening *L. ivanovii* by targeting genes encoding major virulence factors [14]–[16]. However, these approaches might not be suitable for low resource-setting areas because of the high-tech equipment required, elaborate and complicated assay procedures, time requirements and expensive reagents. Therefore, there is a growing demand for simple, economical and easy-operating molecular tests. In this paper, an alternative amplification assay was developed and can be used in the field to detect *L. ivanovii* in the absence of a thermal cycler.

Loop-mediated isothermal amplification (LAMP) is a novel nucleic acid amplification method, known as a rapid, accurate, and cost-effective technique, which had been reported and applied in the field of bacteriological detection [17]–[19]. LAMP contains a set of two specially designed inner (FIP and BIP) and outer (F3 and B3) primers [20]. Two additional primers (loop F and loop B) can be added to the reaction mixture as they increase the sensitivity of the reaction and accelerate the LAMP reaction speed, then can reduce the reaction time by half [18]. Therefore, a LAMP assay requires the recognition of at least six conserved regions more than 180 base pairs [18]. LAMP, using a DNA polymerase such as *Bst* DNA polymerase to induce auto-cycling strand displacement of the target, can be conducted to amplify up to 10^9^ target DNA copies under isothermal conditions (60°C–65°C) within one hour and results can be observed by a visual assessment of turbidity [21], [22].

The current study was to establish a simple, rapid and efficient testing method for detection of *L. ivanovii* by using LAMP assay based on a special identifiable target *smcL* gene and evaluate the diagnostic specificity, sensitivity and suitability of the assay using a panel of bacterial DNA samples and pathogen-simulated human stool. Furthermore, the LAMP assay was compared with qPCR and PCR assays by determining the limit of detection (LoD) levels.

## Materials and Methods

### Ethical considerations

Feces samples were acquired with the written informed consent from a healthy donor. This study was reviewed and approved by the ethics committee of the National Institute for Communicable Disease Control and Prevention, China CDC, according to the medical research regulations of the Ministry of Health, China (Approval No. ICDC-2014003).

### Bacterial strains and culture conditions

Bacterial strains used in this study included 17 *L. ivanovii* and 77 non-*L. ivanovii* strains, as described in Table 1. All the strains were cultured on brain heart infusion agar overnight at 37°C. Strain ATCC BAA-678 was used as a positive control to determine the optimal conditions for LAMP and to establish baseline specificity and sensitivity.

**Table 1:** Bacterial strains used in this study.

Bacteria	Strain no. (source of strain)^a^	No. of strains
*L. ivanovii*	ATCC BAA-678	1
	Isolated strains (ICDC)	16
*L. monocytogenes*	EGDe	1
	NCTC 10890	1
	ATCC 19114	1
	Isolated strains (ICDC)	30
*L. innocua*	ATCC BAA-680	1
	Isolated strains (ICDC)	2
*L. grayi*	ATCC 25402	1
	Isolated strains (ICDC)	5
*L. welshimeri*	ATCC 35897	1
	Isolated strains (ICDC)	8
*L. seeligeri*	ATCC 35967	1
	Isolated strains (ICDC)	1
*Yersinia enterocolitica*	ATCC 23715	1
*Pseudomonas aeruginosa*	ATCC 15442	1
*Enterococcus faecalis*	ATCC 35667	1
*Aeromonas hydrophila*	ATCC 7966	1
*Enterobacter sakazakii*	ATCC 51329	1
*Campylobacter jejuni*	ATCC 33291	1
*Bacillus cereus*	Isolated strains (ICDC)	2
*Salmonella typhimurium*	Isolated strains (ICDC)	4
*Vibrio cholera*	Isolated strains (ICDC)	3
*Escherichia coli*	Isolated strains (ICDC)	9

a ATCC, American Type Culture Collection; NCTC, National Collection of Type Cultures; ICDC, National Institute for Communicable Disease Control and Prevention, China CDC.

### Genomic DNA extraction

Genomic DNA of *Listeria* strains, other common Gram-positive and Gram-negative bacterial strains in pure cultures were extracted with a DNA extraction kit (QIAamp DNA minikits; Qiagen, Hilden, Germany) according to the manufacturer’s instruction. The genomic DNA of *L. ivanovii* strains in artificial contamination human feces were extracted with a QIAamp DNA stool mini kit (QIAGEN, Venlo, Netherlands).

### Design of LAMP Primers

**Figure 1:**
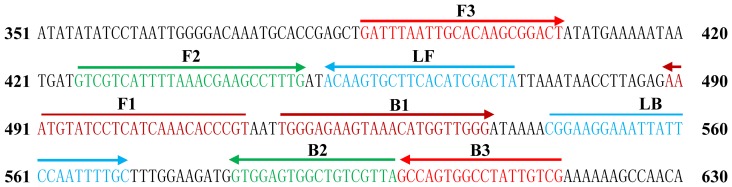
Location and sequences of *L. ivanovii smcL* (*LIV_1186*) gene used to design the six primers. The sequences of the primer sites are underlined. Right and left arrows indicate sense and complementary sequences that are used.

Based on the *smcL* (*LIV_1186*) sequence of *L. ivanovii* (Genbank number: NC_016011.1), more than 30 LAMP primer sets were designed by using Primer Explorer V4 software (http://primerexplorer.jp/). A set of 6 primers was selected for LAMP to target 8 distinct regions in *smcL* after checking the specificity of primer sequences using NCBI BLAST (Basic Local Alignment Search Tool). The sequences and locations of each primer are shown in Table 2 and Fig. 1.

**Table 2:** LAMP and qPCR primers used in this study.

Assay	Primers	Sequence (5′-3′)	Gene location (bp)
*smcL*_LAMP	F3	GATTTAATTGCACAAGCGGACT	391–412
	B3	CGACAATAGGCCACTGGC	608–625
	FIP	ACGGGTGTTTGATGAGGATACATTT-GTCGTCATTTTAAACGAAGCCTTTG	(441–465) (397–421)
	BIP	TGGGAGAAGTAAACATGGTTGGG-TAACGACAGCCACTCCAC	(490–507) (424–446)
	LF	TAGTCGATGTGAAGCACTTGT	468–488
	LB	CGGAAGGAAATTATTCCAATTTTGC	553–557
*smcL*_qPCR	F	ACCCGTAATTGGGAGTAAACAT	518–529
	Probe	HEX-CAGCCACTCCACCATCTTCCAAAGCA-BHQ	574–599
	R	TGTTGGCTTTTTTCGAGAATAGG	608–640

### The LAMP assay

Amplification reactions mixtures of LAMP were performed with the Loopamp Kit (Eiken Chemical Co. Ltd., Tokyo, Japan) in a final volume of 25 µl containing 1.6 mM FIP and BIP primers (each), 0.8 mM LF and LB primers (each), 0.2 mM F3 and B3 primers (each), 12.5 µl 2× reaction mix, 1 µl of *Bst* DNA polymerase (8 U), 1 µl Loopamp Fluorescent Detection Reagent (FD) and 1 µl DNA template. The mixture was incubated at 64°C for 1 h and then at 80°C for 5 min to stop the reaction. Mixture without DNA template was used as a negative control.

A total of 3 methods were used to confirm LAMP DNA amplification. The color change of positive amplification can be directly observed by FD and the positive products were detected by 2% agarose gels electrophoresis with ethidium bromide staining. Moreover, the LAMP products can be monitored by measuring the increased turbidity in real-time using Loopamp Real-time Turbidimeter LA-320C (Eiken Chemical Co., Ltd, Japan).

### Analytical specificity and sensitivity of the *L. ivanovii* LAMP assay

To determine the specificity of the LAMP assay, the LAMP reaction was performed under the conditions described above with DNA templates from 17 *L. ivanovii* strains and 77 non-*L. ivanovii* strains (Table 1). The examinations were carried out in triplicate independently.

In order to compare the sensitivities of the LAMP, qPCR and PCR assays, template DNA from strain ATCC BAA-678 was serially diluted. The LoD of LAMP, PCR and qPCR assays were ascertained by both minimal CFU of bacteria and DNA amount of the template. PCR was performed in 25 µl volume of reaction mixture containing 10 mM Tris-HCl (pH 8.3), 50 mM KCl, 1.5 mM MgCl_2_, 0.001% gelatin, 0.2 µM each of F3 and B3 primer, 0.2 mM each of dNTPs, 1 µl DNA template, and 0.5 units of Taq DNA polymerase (ExTaq; Takara). The program consisted the initial denaturation of 5 min at 95°C, 32 cycles of 30 s at 95°C, 30 s at 56°C and 35 s at 72°C, and a final 5 min extension at 72°C. PCR products were electrophoresed to verify the presence of the expected 235 bp bands. The primers and probe for qPCR assay were shown in Table 2. qPCR amplification was performed in a 25 µl reaction volume containing 0.3 µM each primer, 0.2 µM probe, 1×Premix (Takara Bio, Inc., Otsu, Japan) Ex Taq, and 1 µl of DNA template. The assays were conducted using the PCR settings of pre-denaturation at 95°C for 30 s, 40 cycles of denaturation at 94°C for 5 s, and extension at 60°C for 33 s in an ABI PRISM system (Applied Biosystems, Carlsbad, CA, US). Fluorescence readings were acquired using the 6-carboxyfluorescein (FAM) channel.

### LAMP application in artificial contamination human feces

Human stool samples, which were negative amplification of *L. ivanovii* DNA by traditional culture assay and PCR, were artificially contaminated with *L. ivanovii* strain ATCC BAA-678 at five concentrations (8×10^0^, 8×10^1^, 8×10^2^, 8×10^3^ and 8×10^4^ CFUs per gram), individually [14]. Aliquots (0.2 g) of the stools were used for DNA extraction. This experiment was performed in triplicate independently, and the supernatants (1 µl) were used for LAMP and qPCR detection.

### LAMP application for rapid diagnosis

The detection limit of the LAMP assay in combination with an enrichment step in human stool was also determined in this study. The artificial contaminative samples, which were 500 milligram human stool containing eight CFU of strain ATCC BAA-687, were transferred into 5 ml of Half Fraser’s broth (Oxoid, Hampshire, UK) and homogenized, followed by incubation at 30°C for 2, 4, 6, 8, 10, 12, 14 and 24 h. Aliquots (1 ml) of the enrichment broth were subjected to extracted DNA and used as a template (2 µl) in the LAMP and qPCR assays, which were repeated twice for each sample.

## Results

### Detection and confirmation of *L. ivanovii* LAMP Products

**Figure 2:**
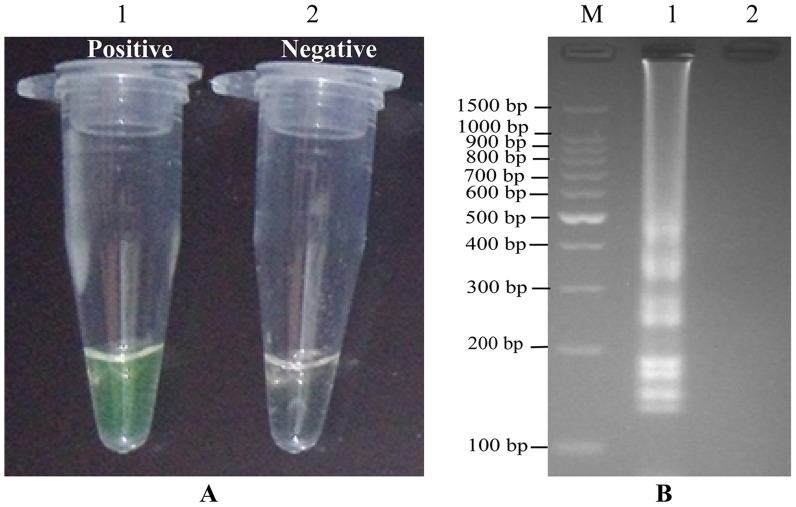
Result of the LAMP on detection of *L. ivanovii* ATCC BAA-678. (A) Color change of the LAMP; Tube 1 positive amplification; Tube 2 negative amplification. (B) 2% agarose gel electrophoresis of LAMP product; Lane M, DNA marker DL100 bp; Lane 1, LAMP product of *L. ivanovii*; Lane 2, negative control.

The color change of positive tubes from light gray to green can be directly observed by naked eyes within a 60-min incubation period (Fig. 2A). The LAMP products were also detected by 2% agarose gel electrophoresis, the specific ladder of multiple bands was visual (Fig. 2B).

### Specificity of the *L. ivanovii* LAMP Assay

To test the specificity of the LAMP primers, the LAMP assay was performed under the conditions described above with DNA templates from 17 *L. ivanovii* strains and 77 non-*L. ivanovii* strains. The reaction tubes with *L. ivanovii* genomic DNA can be directly observed color change within a 60-min incubation period, and the specific ladder of multiple bands was yielded by 2% agarose gel electrophoresis. In the reaction of non-*L. ivanovii* strains, there was no color change in the reaction tubes after a 60-min incubation period and the specific ladder of multiple bands was not produced by 2% agarose gel electrophoresis. This result demonstrated that the LAMP primers were specific to *L. ivanovii* identification.

### Sensitivity of the *L. ivanovii* LAMP assay

**Figure 3:**
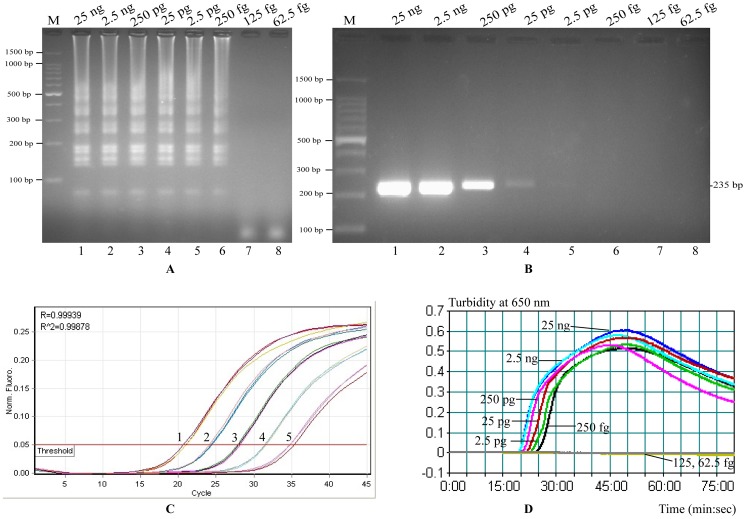
Comparison of the detection limits of the LAMP, PCR and qPCR assays. (A) and (B) Lane M, DNA marker DL100; Lanes 1–8, the results of LAMP and PCR for 25 ng, 2.5 ng, 250 pg, 25 pg, 2.5 pg, 250 fg, 125 fg and 62.5 fg per reaction, respectively. (A) Detection limit of the LAMP reaction (250 fg/reaction); (B) Detection limit of the PCR (25 pg/reaction); (C) Detection limit of the qPCR (2.5 pg/reaction), signals 1–5, the results of qPCR for 25 ng, 2.5 ng, 250 pg, 25 pg, 2.5 pg DNA per reaction, respectively; (D) Real-time sensitivity of *smcL*-LAMP as monitored by the measurement of turbidity (optimal density at 650 nm), the threshold value was 0.1 and a turbidity of>0.1was considered to be positive for *smcL*-LAMP, the detection limit was 250 fg/tube.

Sensitivity of LAMP reaction on *L. ivanovii* was tested by analyzing products yielded from the serial dilutions (25 ng, 2.5 ng, 250 pg, 25 pg, 2.5 pg, 250 fg, 125 fg and 62.5 fg per microliter). The LoD of LAMP, PCR and qPCR assays for *smcL* gene with pure cultures were 250 fg DNA/reaction, 25 pg DNA/reaction and 2.5 pg DNA/reaction, respectively (Fig. 3A, 3B, 3C). This result indicates that the sensitivity of the LAMP assay was 100- and 10-fold more sensitive than that of the PCR and qPCR assay for detecting *L. ivanovii* DNA. Moreover, the LAMP reaction required 19-, 20-, 23-, 25-, 27- and 29-min incubation periods at six levels of genomic DNA (25 ng, 2.5 ng, 250 pg, 25 pg, 2.5 pg and 250 fg per reaction), respectively (Fig. 3D).

### Evaluation of LAMP assay in simulated human fecal specimens

To demonstrate the utility of the LAMP method as a surveillance tool for *L. ivanovii*, the artificial contamination of human fecal specimens were analyzed by the LAMP and qPCR assays. The LAMP assays detected the presence of *L. ivanovii* strains down to as little as 8×10^3^ CFU/g in simulated human stool samples. By contrary, the qPCR assays had a detection limit of 8×10^4^ CFU/g for *smcL* gene in artificial contamination of human fecal specimens (data not shown).

### The LAMP application for rapid diagnosis

**Figure 4:**
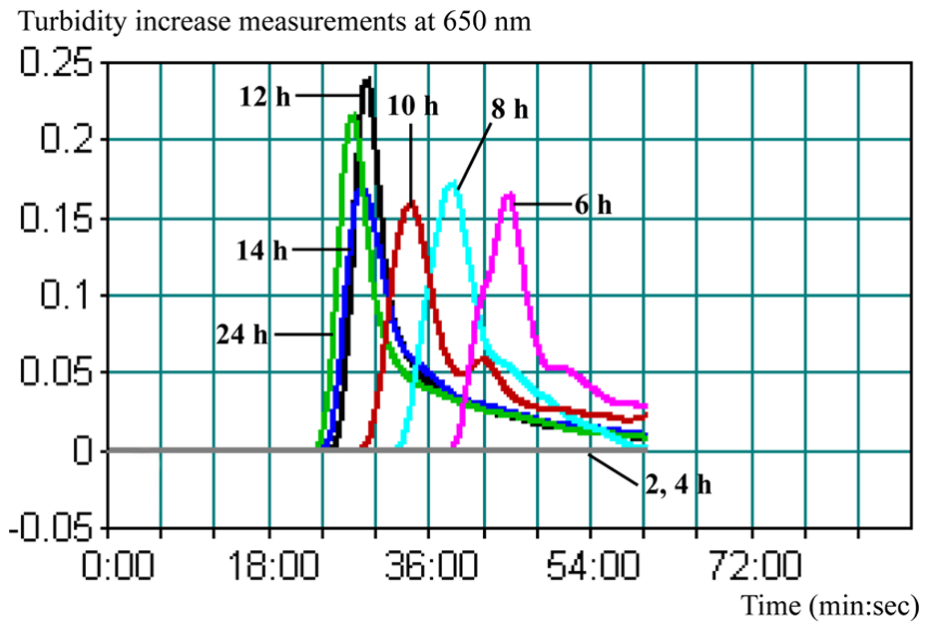
The LAMP amplification graph obtained when testing human fecal specimens spiked with the low level of *L. ivanovii* strain after various enrichment periods (2, 4, 6, 8, 10, 12, 14 and 24 h) in Half Fraser’s broth. The human fecal specimen was spiked with 8 CFU/0.5 g of *L. ivanovii* strain ATCC BAA-678.

The capability of the LAMP assay to rapidly detect low levels of *L. ivanovii* in human fecal specimens was also evaluated. The human fecal specimens, which were contaminated with *L. ivanovii* strains at level of 8 CFU/0.5 g, were cultured in Half Fraser’s broth after different enrichment periods. A typical LAMP judgment graph generated for human stool enrichment samples was shown in Fig. 4. None of 2- and 4-h enrichment samples tested positive for *L. ivanovii* by either qPCR or LAMP assay. The positive LAMP reactions were observed with 6-, 8-, 10-, 12-, 14- and 20-h and the turbidity of >0.1 was considered to be positive. Moreover, the minimum positive reaction times were 40, 34, 30, 25, 25, 25 min for 6, 8, 10, 12, 14 and 20 h enrichment samples, respectively. A similar trend of detection was observed for qPCR. However, the qPCR results were presented by cycles, which were approximately 1 min/each. Therefore, an additional 20–40 min of reaction time was needed for qPCR when testing the same enrichment sample.

## Discussion

*L. ivanovii* is the only other pathogenic species of the genus *Listeria*, and is particularly associated with abortion in ruminants, but is also responsible for rare cases of human listeriosis [23], [24]. However, *L. ivanovii* occurrence can be locally highly prevalent and the pathogen has been isolate form food products, environment, healthy animal and human carriers, thus the contribution of *L. ivanovii* to human cases may have been underestimated [10], [25]. Therefore, rapid identification, efficient prevention and control strategies for *L. ivanovii* are extremely important from a public health perspective.

The traditional culture-based methods for *L. ivanovii* detection, which involves enrichment, colony formation on selective agar medium, morphological and biochemical tests, is tedious and lengthy, making it difficult to rapidly detect causative pathogens associated with sporadic and outbreaks cases [26]. PCR assay, developed to rapidly detect the target pathogen in enrichment cultures, generally requires gel electrophoresis for determining results, which has poor sensitivity, and is labor-intensive and time-consuming [14]. Modified PCR assays, such as real-time and nested PCR, are complicated and require a high-precision thermal cycler, and therefore, the application of PCR-based assays were limited to diagnose *L. ivanovii* in basic clinical, microbiology and field laboratories [27].

In order to simply, rapidly and accurately identify *L. ivanovii*, we developed a LAMP assay for sensitive and specific detection of the pathogen targeting a conserved region of the *smcL* gene, which encodes sphingomyelinase C, a membrane-damaging virulence factor in *L. ivanovii* [28]–[30]. This is the first time to report a LAMP method for identifying *L. ivanovii*, which can be used in field, medical and veterinary laboratories. The established LAMP assay does not require gel electrophoresis, expensive equipment and trained personnel, only maintaining a constant temperature of 64°C for 1 h is sufficient for the reaction. The LAMP assay detected as little as 250 fg genomic DNA per reaction in pure cultures, and were 10- and 100-fold more sensitive than the qPCR and conventional PCR method. Moreover, the strain ATCC BAA-678 was used in experiments with simulated human stool samples, with the LAMP assays having a detection limit of 8×10^3^ CFU/g for the stool, which was also 10 times more than the qPCR assay. Thus, the LAMP assay is more suitable than qPCR and PCR methods for simple, rapid and sensitive detection of *L. ivanovii*.

A total of 94 strains, which consisted of 17 *L. ivanovii* and 77 non-*L. ivanovii* strains, were used for the inclusivity and exclusivity tests, respectively. The positive amplifications were obtained from 17 *L. ivanovii* strains, and negative reactions were observed in the assay of non-*L. ivanovii* strains. The positive amplification can be done by visual inspection within 1 h, and the positive results were also detected by agar gel electrophoresis or real-time measurement of turbidity. Therefore, lower detection limit, less reaction time and the high specificity of the LAMP assay targeting *smcL* gene poses an advantage for broad application in listeriosis surveillance and public health.

The artificial contaminative human feces with low level of *L. ivanovii* strains were used to evaluate the capability of the established LAMP assay for rapidly diagnosing this pathogen. Positive detection, only required once enrichment in Half Fraser’s broth, occurred for the human stool samples spiked with 8 CFU/0.5 g of ATCC BAA-678 after a 6-h period of enrichment. Moreover, our LAMP assay was superior to qPCR with respect to LoD and assay speed in artificial contamination of human stool samples, and proved markedly faster than qPCR by at least 20 minutes. Therefore, the LAMP technique is more sensitive, accurate and rapid than qPCR methods for the detection of *L. ivanovii* in simulated human stool samples, and valuable for the application in foodbrone contamination and listeriosis diagnosis caused by *L. ivanovii.*

In conclusion, we developed a novel LAMP assay targeting *smcL* gene for detection of *L. ivanovii*, which was simple, rapid, sensitive, specific and economical. Thus, the LAMP assay may facilitate rapid and reliable diagnosis of *L. ivanovii* in field, medical and veterinary laboratories.
